# Primary breast angiosarcoma: A case report

**DOI:** 10.1097/MD.0000000000039186

**Published:** 2024-08-02

**Authors:** Yan Wang, Shengrong Xie, Dawei Peng, Jin Zhou, Shuangye Hu

**Affiliations:** a Department of Breast Surgery, Longquanyi District of Chengdu Maternity and Child Health Care Hospital, Chengdu, China; b Department of Orthopaedics, The First People’s Hospital of Longquanyi District, Chengdu City, Sichuan, China; c Department of Radiology, Longquanyi District of Chengdu Maternity and Child Health Care Hospital, Chengdu, China; d Chengdu University of Traditional Chinese Medicine, Chengdu, China; e Department of Pathology, Longquanyi District of Chengdu Maternity and Child Health Care Hospital, Chengdu, China.

**Keywords:** breast swelling, breast trauma, primary breast angiosarcoma

## Abstract

**Rationale:**

Primary breast angiosarcoma is a rare tumor, accounting for only 0.05% of all malignant breast tumors. The primary breast angiosarcoma typically presents with nonspecific clinical manifestations, which can easily lead to misdiagnosis. Potential factors contributing to misdiagnosis include skin changes that may be erroneously attributed to breast trauma-induced bruising and breast swelling that may be mistaken for inflammatory diseases or other benign tumors.

**Patient concerns:**

A 19-year-old female was admitted to the hospital due to repeated lump formation in the left breast for 9 months after left breast trauma.

**Diagnoses:**

The diagnosis of primary breast angiosarcoma was confirmed on hematoma biopsy.

**Interventions:**

Due to the patient’s condition, no special treatment was given postoperatively. After then, there was a recurrence in the chest wall, and the patient received 2 cycles of chemotherapy, resulting in a reduction in the size and lightening of the recurrent chest wall mass. When chemotherapy intolerance happened, the patient chose to discontinue treatment.

**Outcomes:**

After an 18-month follow-up, the recurrent chest wall mass increased and the patient died from bleeding.

**Lessons:**

Primary breast angiosarcoma has a low incidence but high malignancy, with a high recurrence and metastasis rate, leading to a poor prognosis. The adjuvant chemotherapy, radiotherapy, targeted therapy, and other treatments should be considered to reduce the local recurrence rate and prolong patient survival.

## 1. Introduction

Breast angiosarcoma (BA) is a rare tumor that originates from the endothelial cells of the capillaries or lymphatic vessels surrounding the lobular tissue of the breast. It accounts for only 0.05% of all malignant breast tumors^[1]^ and can be classified into primary BA (PBA) and secondary BA. PBA tends to affect younger women aged 20 to 40 years, and its pathogenesis remains unclear, though it might be related to overexpression of hypoxia-inducible factor-1α, vascular endothelial growth factor, and Wilms tumor-1 protein.^[2,3]^ On the other hand, secondary BA is more prevalent in older women and is often associated with a history of breast-conserving surgery and radiation therapy or chest wall radiation therapy.^[4]^ The aggressive nature of angiosarcoma, marked by rapid proliferation and infiltrative growth, leads to high rates of metastasis and postoperative recurrence, with an average natural course lasting only 1.1 to 2.8 years.^[5]^ This study retrospectively analyses clinical data from a single case of PBA admitted to our department, aiming to delve deeper into the diagnosis, treatment, and prognostic factors of PBA.

## 2. Case presentation

**Figure 1.**
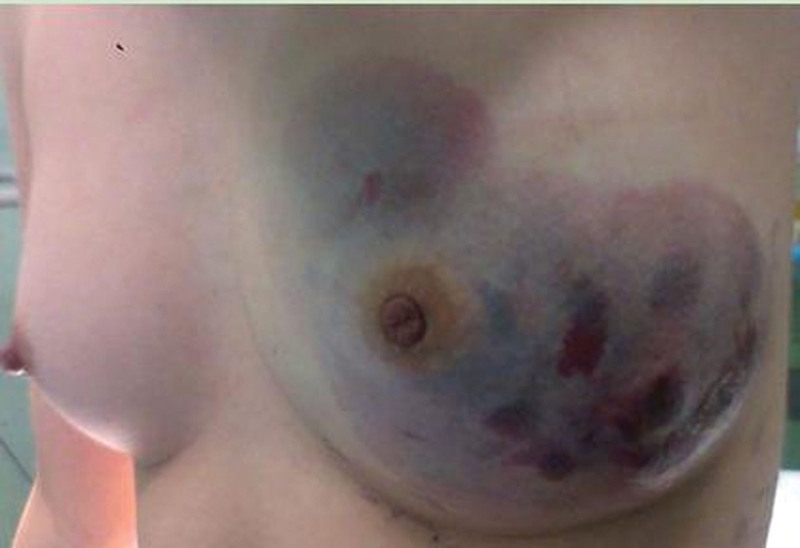
Preoperatively, the left breast exhibited significant enlargement, accompanied by elevated skin surface tension and extensive purplish discoloration surrounding the areola.

**Figure 2.**
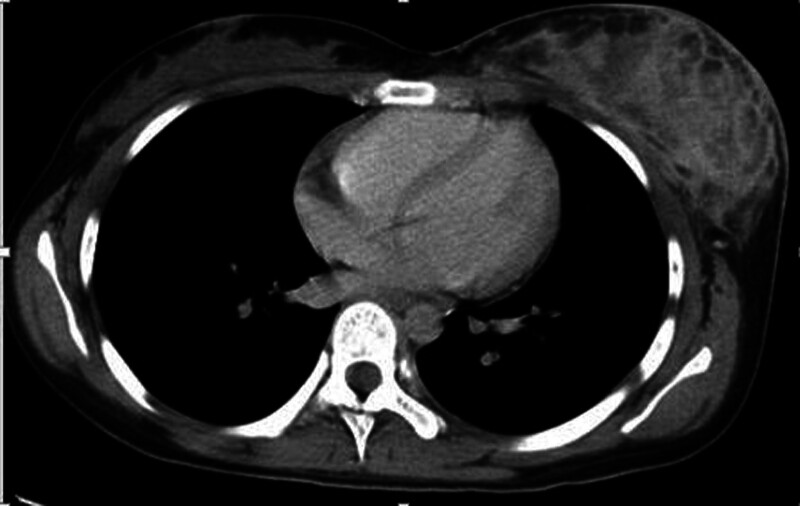
The enhanced computed tomography (CT) scan of the chest revealed multiple cystic abnormal density shadows in the lower outer quadrant of the left breast, exhibiting heterogeneous enhancement and dermal thickening.

A19-year-old female was admitted to the hospital due to recurring lump formations in her left breast for 9 months following trauma to the same breast. Initially, she experienced bruising and swelling, undergoing hematoma removal surgery twice outside the hospital, resulting in temporary relief from swelling. However, the bruising and swelling recurred and gradually worsened, leading to hospital admission. There was no history of similar clinical manifestations or cancer in the patient’s family. On physical examination, notable enlargement of the left breast was observed, accompanied by a large blue-purple discoloration of the skin around the lower outer quadrant and areola (Fig. 1), with evident edema. Palpation revealed a soft, indistinctly bordered, tender mass measuring approximately 8.0 × 5.0 cm within the left breast. No enlarged lymph nodes were detected in the corresponding axilla or supraclavicular regions. Breast ultrasound exhibited thickened skin and subcutaneous tissue in the left breast, along with a hypoechoic mass displaying a relatively clear boundary in the lower outer quadrant, accompanied by an irregular dark area exhibiting abundant blood flow signals. Additionally, an enhanced chest computed tomography scan (Fig. 2) revealed multiple cystic abnormal density shadows in the left breast, featuring uneven enhancement, obvious enhancement of the cyst wall and septum, and low-density cystic fluid lacking enhancement in the center. The left breast skin was also thickened. Aspiration from the mass yielded a viscous red bloody fluid.

**Figure 3.**
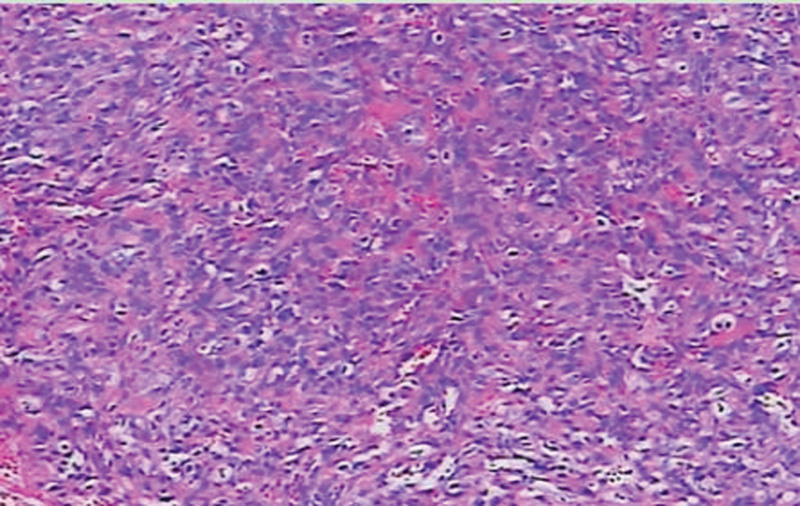
The endothelial cells exhibited clustered proliferation upon HE staining, while the epithelial cells displayed spindle-shaped and multi-angular morphology with significant atypia. (×200).

**Figure 4.**
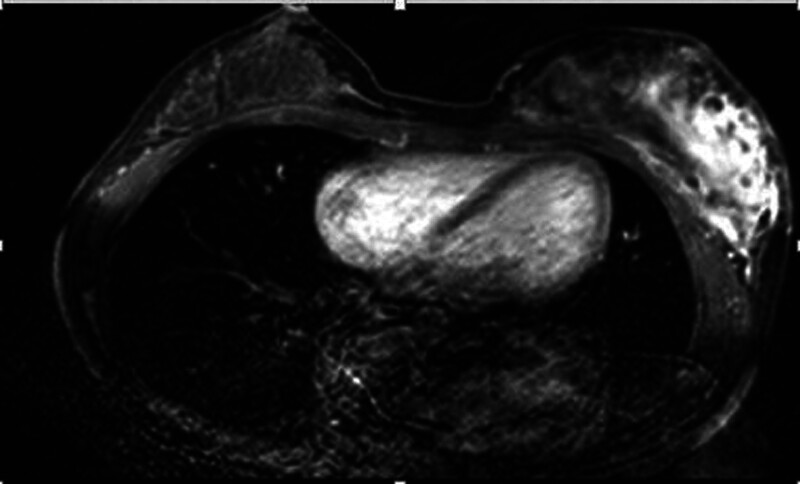
Enhanced MRI revealed pronounced heterogeneous nodular enhancement in the left breast, accompanied by infiltration of the left chest wall and thickening of the adjacent cutaneous tissue.

**Figure 5.**
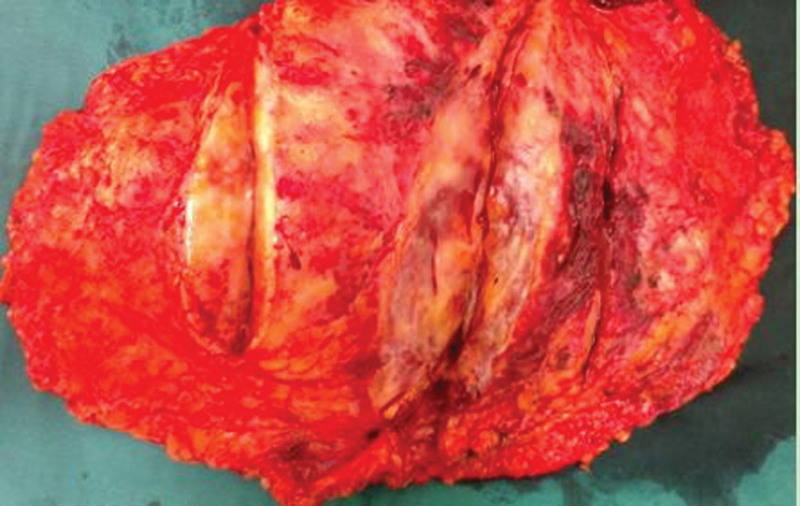
The specimen obtained from the simple mastectomy of the breast exhibited a solid tumor lacking a distinct capsule, indistinct boundaries, and a cut surface displaying a dark red fish-like appearance.

**Figure 6.**
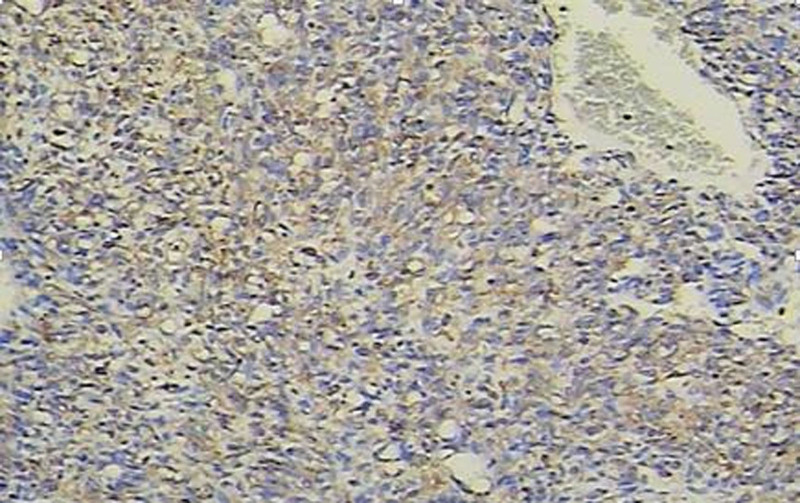
The tumor cells demonstrated positive immunoreactivity for CD31 via immunohisto chemistry (IHC) (×200).

**Figure 7.**
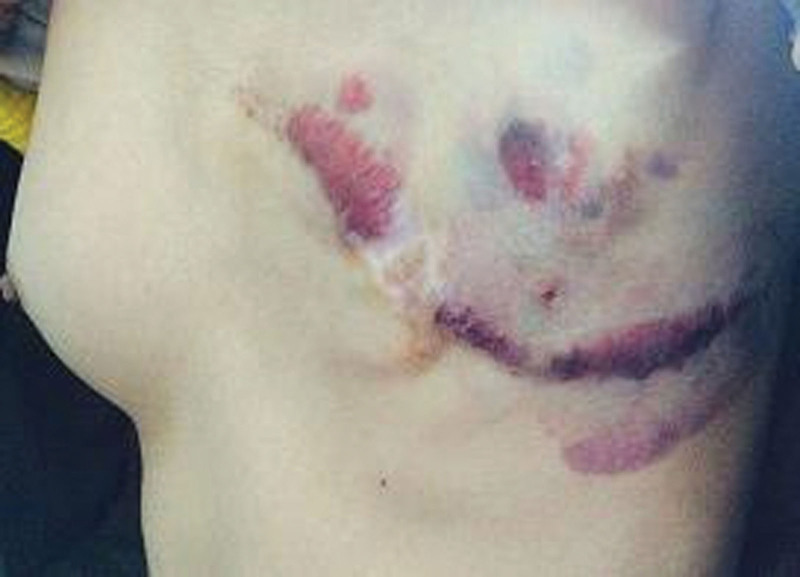
After a period of 10 months following the surgical procedure, there was an observed recurrence of the incision and chest wall, characterized by localized purplish discoloration of the skin.

**Figure 8.**
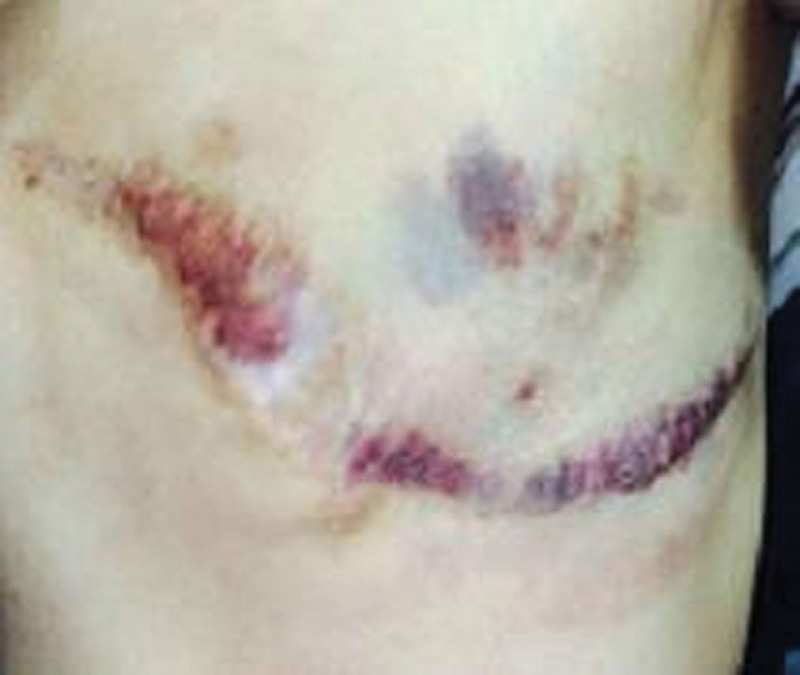
After undergoing 3 cycles of chemotherapy, the tumor in the chest wall exhibited a reduction in size, while the localized purplish discoloration demonstrated notable improvement.

Initial assessment suggested a left breast hematoma with potential tumor rupture and bleeding. Following hematoma removal and biopsy, postoperative pathology (Fig. 3) revealed left breast angiosarcoma (grades 2 to 3). Five days after surgery, enhanced breast magnetic resonance imaging (MRI) (Fig. 4) indicated extensive areas displaying prolonged T1 and T2 signal shadows, high signal on SPectral Attenuated Inversion Recovery, slightly elevated signal on diffusion-weighted imaging, marked irregular enhancement, nodular enhancement upon contrast-enhanced scanning, multiple round low signal shadows internally, infiltration of the left chest wall, and adjacent skin thickening. The diagnosis of left breast angiosarcoma with chest muscle infiltration prompted a second surgery involving unilateral mastectomy and partial resection of the pectoralis major muscle. Intraoperatively, the tumor, approximately 10.0 × 8.0 cm in size, was deep within the breast tissue, solid, lacking a distinct capsule, displaying unclear borders, and a fish-flesh-like appearance upon sectioning, with infiltration into the pectoralis major muscle (Fig. 5). Microscopic analysis (Fig. 3) revealed the tumor comprising interlacing vascular lumens, notable endothelial cell atypia, spindle-shaped and multi-angular epithelial cells forming a solid tumor area, exhibiting mitotic figures, areas of hemorrhagic necrosis, and invasion into the pectoralis major muscle. Immunohistochemistry indicated positivity for cluster of differentiation (CD) 31 (Fig. 6) and CD34 and focal positivity for factor VIII. Postoperatively, no specific treatment was administered due to the patient’s condition. However, 10 month laters, recurrence in the chest wall (Fig. 7) prompted 2 cycles of ifosfamide and cisplatin chemotherapy, resulting in size reduction and fading of the recurrent chest wall mass (Fig. 8). Subsequently, due to chemotherapy intolerance, the patient discontinued treatment. Regrettably, over an 18-month follow-up, the recurring chest wall mass progressed, culminating in the patient’s demise from bleeding.

## 3. Discussion

This paper reviews a case of primary breast angiosarcoma that was misdiagnosed as breast hematoma, and summarizes the disease characteristics and key points of diagnosis and treatment of breast angiosarcoma based on the domestic and international literature, in order to provide clinical reference for diagnosis and treatment, and avoid misdiagnosis.

The diagnostic challenge of PBA stems from its nonspecific clinical presentation and auxiliary examination findings, leading to notably high misdiagnosis rates.^[6,7]^ Typically, this condition manifests as a rapidly enlarging breast mass accompanied by acute pain. The skin over the mass might exhibit purplish-blue or purplish-red discoloration. In more extensive tumors, it can induce Kasabach–Merritt syndrome, causing thrombocytopenia and bleeding.^[6]^ Skin changes can easily cause misdiagnosis and may be mistaken for congestion caused by breast contusion. In this case, bleeding was considered to be caused by contusion after 2 treatments outside the hospital, which delayed the diagnosis and led to the progression of the disease. Therefore, when the skin color change is encountered and the lesion recurs or progresses after treatment, biopsy is needed to confirm the diagnosis.

Neither breast ultrasound nor mammography offers specific diagnostic clues.^[8]^ MRI scans often reveal intratumoral hemorrhage, early peripheral enhancement on dynamic contrast-enhanced imaging, and subsequent expansion of the enhancement zone over time. This examination is crucial for delineating lesion extent and characteristics, crucial for precise diagnosis and treatment planning.^[9]^ Vascular sarcomas commonly express endothelial markers such as CD31 and/or CD34, although poorly differentiated might show loss of expression. Erythroblast transformation-specific-related gene and friend leukaemia virus integration gene 1 are emerging markers for breast angiosarcoma, demonstrating superior sensitivity and specificity compared to CD31 and CD34.^[10,11]^

Currently, surgical treatment stands as the primary approach for PBA. However, due to its rarity and limited clinical cases along with the absence of prospective studies and randomized controlled trials, determining the optimal surgical strategy for patients remains unclear. Common surgical methods include local excision, wide excision, and mastectomy. While some studies indicate that partial or total mastectomy does not significantly affect patient survival time,^[12,13]^ other studies present conflicting outcomes, suggesting that patients undergoing breast-conserving surgery experience longer overall survival.^[14,15]^ This discrepancy might stem from the fact that patients undergoing breast-conserving surgery have smaller tumors, an earlier clinical stage, and a higher likelihood of achieving negative margins. PBA diagnosis frequently occurs at advanced stages with extensive tumor infiltration, posing challenges in attaining negative margins through wide local excision, resulting in a heightened postoperative local recurrence rate. Currently, mastectomy with negative margins presents the standard surgical procedure. Moreover, since angiosarcomas originate in stromal tissue and primarily spread via the bloodstream, lymph node involvement is uncommon, leading to infrequent axillary lymph node dissection.^[16]^ Naturally, in clinical practice, the choice of surgical approach depends on various factors such as patient preferences, tumor size, breast volume, and the extent of lesion infiltration.^[17]^ In this case, extensive tumor infiltration beyond the palpable mass during surgery led to the belief that unilateral mastectomy would comprehensively eradicate the lesion, minimize residual disease, and reduce recurrence risk.

The role of adjuvant therapy in managing PBA lacks international clarity, leading to no consensus on chemotherapy regimens. Certain studies highlight docetaxel’s potent inhibitory effect on angiosarcoma proliferation, while paclitaxel-based chemotherapy has shown enhanced survival rates.^[18]^ In cases of metastatic angiosarcoma, treatment involving doxorubicin, paclitaxel, and cisplatin has proven effective,^[19]^ with paclitaxel-based regimens being preferred by many clinicians. Alongside surgery and chemotherapy, radiation therapy stands as a widely used treatment method. Adjuvant radiotherapy reduces recurrence rates, particularly in cases with microscopically positive margins (R1) after resection.^[20]^ Research indicates a 5-year local recurrence-free interval of 57% with surgery plus radiotherapy compared to 34% with surgery alone.^[21]^ Furthermore, most angiosarcomas express vascular endothelial growth factor receptors, and targeting these receptors can impede tumor angiogenesis, indirectly inhibiting tumor growth and infiltration, and reducing metastasis incidence.^[22]^ Recent clinical trials exploring targeted therapies such as pembrolizumab in combination with axitinib provide promise for angiosarcoma patients less responsive to chemotherapy and radiation therapy.^[23]^ However, due to the lack of large-scale case studies, the clinical application of targeted therapy for angiogenesis is limited, necessitating further research.

The primary causes of mortality postoperatively in PBA involve early onset, poor prognosis, and the likelihood of recurrence or metastasis. Current research indicates a median survival time of 38 months, with 1-, 3-, and 5-year overall survival rates of 80%, 39%, and 25% respectively.^[14]^ Prognostic factors identified include tumor histological grade, staging, size, and surgical margins.^[15,24]^ In this case, the patient exhibited poor histological differentiation, a large tumor infiltrating the pectoralis major muscle, an early disease onset, and recurrence within 10 months postoperatively, ultimately succumbing to hemorrhage from tumor recurrence at 18 months. Given the non-specificity of clinical presentation and diagnostic imaging such as breast ultrasound and mammography, coupled with significant morphological variations in pathological tissue, accurately differentiating PBA from other breast diseases is crucial. In well-differentiated cases, differentiation is essential from benign vascular tumors, angiolipoma, and pseudoangiomatous stromal hyperplasia. In poorly differentiated cases, differentiation is essential from breast adenocarcinoma, malignant phyllodes tumor, malignant melanoma, fibrosarcoma, and solitary fibrous tumor.^[25]^

In addition, the present study also had limitations. Genetic changes in cancer cells can guide cancer therapy, however, here the cancer tissue was not subjected to DNA sequencing to further identify potential gene mutations or genetic polymorphism. Moreover, after the surgery, the patient refused adjuvant treatments such as chemotherapy. When the cancer recurred later, the patient only received 2 cycles of chemotherapy but was unable to tolerate it, and subsequently abandoned further treatment.

## 4. Conclusions

Primary breast angiosarcoma, though rare, exhibits high malignancy, accompanied by elevated recurrence and metastasis rate, resulting in an unfavorable prognosis. Prolonged nonhealing following breast trauma and rapid lesion enlargement with bluish-purple discoloration should prompt suspicion, necessitating differentiation from traumatic hematoma and vascular tumors. Our recommendation includes conducting an enhanced MRI and biopsy for early diagnosis, followed by complete surgical excision of the lesion with negative margins. This step proves pivotal in eradicating the lesion and improving the prognosis. Considering adjuvant chemotherapy, radiotherapy, targeted therapy, and other treatments becomes crucial to reduce local recurrence rates and prolong patient survival.

## Author contributions

**Conceptualization:** Jin Zhou, Shuangye Hu.

**Data curation:** Yan Wang, Shengrong Xie, Dawei Peng, Jin Zhou, Shuangye Hu.

**Formal analysis:** Yan Wang.

**Funding acquisition:** Jin Zhou.

**Methodology:** Yan Wang, Shengrong Xie, Dawei Peng, Shuangye Hu.

**Writing – original draft:** Yan Wang.

**Writing – review & editing:** Yan Wang, Shuangye Hu.
